# Opportunities to improve storage and transportation of blood specimens for CD4 testing in a rural district in Zimbabwe using BD vacutainer CD4 stabilization tubes: a stability and diagnostic accuracy study

**DOI:** 10.1186/s12879-014-0553-9

**Published:** 2014-10-22

**Authors:** Emmanuel Fajardo, Carol Metcalf, Elton Mbofana, Charlotte van Vyve, Dhodho Munyaradzi, Sandra Simons, Misheck Kuhudzayi, Helen Bygrave

**Affiliations:** Médecins Sans Frontiéres, Southern Africa Medical Unit, Wyecroft Road, Waverley Business Park, Block 20, Suites 303 A&B, Mowbray, 7925 Cape Town, South Africa; Médecins Sans Frontiéres, Buhera District, Buhera, Zimbabwe; Médecins Sans Frontiéres, Harare, Zimbabwe; Ministry of Health and Child Welfare Zimbabwe, Buhera District, Zimbabwe

**Keywords:** CD4+ T-cell testing, Antiretroviral therapy, Specimen transport, Resource-limited settings, Zimbabwe

## Abstract

**Background:**

CD4+ T-cell testing of blood specimens collected in standard EDTA Vacutainer tubes and transported at ambient temperature, must be completed within 48 hours with the BD FACSCount™ flow cytometer, restricting specimen collection in remote clinics with no on-site testing and limited specimen transport services. We conducted a study in Buhera District, Zimbabwe, to assess the stability and accuracy of CD4+ T-cell results of samples collected in Stabilization Tubes (ST) and stored at ambient temperature for varying time periods.

**Methods:**

Paired EDTA and ST samples were collected from 51 HIV-positive patients aged 18 years and older. CD4+ T-cell testing was done on arrival in the laboratory (Day 0). ST samples were retested on Days 3, 5, and 7. Nineteen ST samples were stored for an additional week and retested on Day 14.

**Results:**

There was a strong correlation between absolute CD4+ T-cell counts measured in the EDTA Day 0 reference sample and Day 7 ST sample (Spearman's rho: 0.9778; mean difference: -4.9 cells/μL and limits of agreement (LOA): 98.5 and 88.7 cells/μL); and the reference sample and Day 14 ST sample (Spearman's rho: 0.9632; mean difference 5.1 cells/μL and LOA: -99.6 and 109.8 cells/μL. Using a 350 cells/μL threshold, the sensitivity, specificity, positive predictive value (PPV) and negative predictive value (NPV) were all 100% on Day 7, and 83.3%, 100%, 100% and 92.9% on Day 14. Using a 500 cells/μL threshold, the sensitivity, specificity, PPV and NVP were 100%, 88.5%, 88.5% and 100% on Day 7 and 88.9%, 80.0%, 80.0% and 88.9% on Day 14.

**Conclusions:**

CD4 ST can be used and stored up to 7 days as a reliable alternative to standard EDTA tubes in settings where CD4+ T-cell testing within 48 hours is not feasible. Despite the small sample size, results suggest that ST may be stored up to 14 days at room temperature for CD4 testing, without compromising accuracy. However, further studies with larger sample sizes are needed to confirm this preliminary finding.

**Electronic supplementary material:**

The online version of this article (doi:10.1186/s12879-014-0553-9) contains supplementary material, which is available to authorized users.

## Background

Among people with HIV infection, CD4+ T-cell count is used to determine eligibility for antiretroviral treatment (ART) and to monitor treatment response. The 2013 World Health Organization (WHO) HIV guidelines recommend that ART be provided to all individuals with confirmed HIV infection who have CD4+ T-cell cell counts ≤500 cells/μL, and that individuals with CD4+ T-cell counts ≤350 cells/μL should be given priority to initiate treatment [1].

In countries without access to routine viral load testing, WHO guidelines stipulate that CD4+ T-cells be measured every six months to monitor response to treatment [1]. In addition to the need of CD4 testing for treatment eligibility and monitoring response to treatment, CD4+ T-cell testing is used to guide initiation of co-trimoxazole prophylaxis in patients with a CD4+ T-cell count ≤200 cells/μL or ≤350 cells/μL to prevent co-infections such as *Pneumocystis carinii*, toxoplasmosis and bacterial infections [2], and the initiation of fluconazole prophylaxis in patients with a CD4+ T-cell count ≤100 cells/μL to prevent cryptococcal disease [3]. These recommendations were adopted by the Zimbabwean government as policy and are contained in the December 2013 Zimbabwean national guidelines [4].

Access to CD4 testing is dependent on having adequate laboratory capacity, and having the means to transport specimens from peripheral health centres to a district or central laboratory [5],[6]. The Beckton Dickinson FACSCount flow cytometer remains one of the most used CD4 testing technologies in resource-limited settings [7]. However, blood specimens collected in standard EDTA tubes and transported at ambient temperature must be completed within 48 hours [8]. Due to this short interval to collect specimens and transport them to the district laboratory, patients are often requested to return to the clinic on specific days of specimen collection and additional visits to the clinic may lead to high rates of pre-ART attrition [9],[10] or failure to perform monitoring tests to detect treatment failure.

Although new point-of-care (POC) CD4 testing technologies have emerged recently, and are considered an important tool to improve retention in care prior to treatment initiation [11], costs may hamper widespread implementation in resource-limited settings [12]. High throughput laboratory-based CD4 testing will therefore remain an important component in a tiered approach to service implementation.

Blood stabilizers that allow for storage of blood specimens for longer periods of time have been developed previously [13],[14]. The BD Vacutainer® CD4 stabilization tubes (ST) (Becton-Dickinson Vacutainer Systems, Franklin Lakes, NJ) enable peripheral blood mononuclear cells (PBMCs) to be preserved for a longer period than with standard blood collection tubes, enabling the interval between blood collection and laboratory testing to be increased without compromising measurement accuracy. According to the manufacturer's instructions, blood specimens collected in BD CD4 ST can be stored at temperatures up to 37°C for up to three days, and at temperatures up to 30°C for up to seven days before CD4 testing. Although BD CD4 ST have been commercially available since 2006 [15] there is only one published study, done in Uganda, that has documented the performance of CD4 stabilization tubes under field conditions using the FACSCount flow cytometer and the anti-CD3 and anti-CD4 antibodies [16]. This study showed an average CD4 cell decline of 6 cells/day (95% CI, 3.6 – 9.0 cells/day) over the first 8 days. The rate of decline was less during the first six days (3 cells/day; 95% CI, 0.5 - 7.4 cells/day) than during the subsequent two days (10 cells/day; 95% CI, 4.6 - 24.9 cells/day) with storage at ambient temperatures between 25°C and 28°C.

Médecins Sans Frontiéres (MSF) has been supporting the HIV/TB programme in Buhera District in Manicaland Province of Zimbabwe since 2004. The programme provides ART and management of opportunistic infections (OIs) in children and adults with HIV infection through an OI clinic at Murambinda Mission Hospital (MMH) and 22 decentralised clinics. In Buhera district, a weekly specimen collection service is provided to the primary health care clinics that provide decentralised ART. This enables assessment of eligibility for ART, and monitoring of response to treatment to be carried out without the need for the patient to travel to the central hospital. However if the patient's follow-up visit does not coincide with the day that specimens are collected, this necessitates an additional clinic visit for the patient.

The use of CD4 STs for specimen collection, instead of EDTA Vacutainer tubes, would decrease the number of patients needing to return to the clinic for specimen collection because blood specimens could be collected on the day of the first consultation and be stored for up to 7 days before CD4+ T-cell testing in the laboratory. Given the lack of evidence on the performance of BD stabilization tubes under field conditions, we conducted a prospective study to assess the performance of CD4 STs for CD4 measurement under field conditions in Buhera District in Zimbabwe.

## Methods

### Study population

Blood samples were obtained from 51 HIV-infected individuals aged 18 years or older who were having blood drawn to determine ART eligibility or monitor response to treatment at any of four MSF-supported primary healthcare clinics in the rural district of Buhera, in Manicaland Province in Zimbabwe. Ethics approval for the study was obtained from the Medical Research Council of Zimbabwe and the Médecins Sans Frontiéres Ethics Review Board. All study participants provided written informed consent to participate in the study and for the collection of a second sample of venous blood in a CD4 Stabilization Tube, which was used as the alternative blood collection method.

### Laboratory procedures

#### Sample collection and storage in the clinics

In each clinic, a nurse obtained venous blood samples from each participant by venipuncture. Venous blood was collected in a standard 4-ml K_2_ EDTA Vacutainer tube, which was used as the reference blood collection method. A second sample of venous blood was collected in a 2-ml CD4 Stabilization Tube [BD Vacutainer; Becton Dickinson Vacutainer Systems, Franklin Lakes, New Jersey, USA] which was used as the alternative blood collection method. Tubes were inverted ten times and were stored in vertical racks and transported at room temperature (18°C - 25°C) to the nearest district laboratory within 8 hours of sample collection.

#### Sample processing, storage and testing in the laboratory

The laboratories processed blood samples within 8 hours of arrival (Day 0). In each laboratory, an experienced laboratory technologist carried out the testing. Absolute CD4+ T-cell counts were determined using the BD FACSCount™ flow cytometer (Becton Dickinson, San Jose, California, USA) and the BD FACSCount™ CD4 Reagent according to the manufacturer's instructions. The BD FACSCount™ CD4 Reagent comprises a single tube which contains all reagents required for absolute CD4+ T-cell counts and CD4+ T-cell percentages. The BD FACSCount™ CD4 Reagent contains three human fluorochrome-labeled monoclonal antibodies, namely, CD4 PE, CD14 PE-Cy™5, CD15 PE-Cy5, which bind specifically to lymphocyte surface antigens, and a fluorescent nuclear dye binds to the nucleated blood cells. In addition, the reagent tubes also contain a known number of fluorescent reference beads. Briefly, 50 μL of anticoagulated venous blood was added to the BD FACSCount™ CD4 reagent tubes by reverse pipetting using the pre-programmed BD FACSCount™ electronic pipette. Reagent tubes were incubated in the workstation for 30 minutes at room temperature (18°C to 25°C). After 30 minutes, fixative solution was added and tubes were then vortexed and analyzed with the BD FACSCount™ flow cytometer. The software automatically identifies lymphocyte populations and calculates the CD4 counts (cells/μL) by comparing cellular events to bead events. At least 10,000 events (20,000 - 45,000 events for the majority of samples) were acquired. Results included CD4 counts and CD4 percentages, and were printed immediately after samples were run.

Internal quality control (IQC) with BD FACSCount Control Kit (BD Biosciences) was carried out daily prior to routine testing, according to standard operating procedure in order to verify system accuracy and linearity. EDTA tubes were discarded after completion of testing on Day 0. ST samples were stored in vertical racks at room temperature and were retested on Days 3, 5 and 7, after inverting the tube at least 10 times. In one laboratory, 19 ST samples were retested on Day 14. Both laboratories were enrolled in the AFREQAS external quality assurance programme (proficiency testing) provided by the National Health Laboratory Service (NHLS) in South Africa. The laboratory technologists who carried out the testing were blind to the reference sample results.

#### Statistical analysis

Data was analysed using Stata 11 (StataCorp, College Station, Texas, USA). ST results at each time-point (index test) were compared with the EDTA Day 0 result (reference test). Simple statistics, including minimum, maximum, median, and interquartile range (IQR) were used to describe the distribution of test results by specimen type and day of testing. Correlation between the ST sample result and the reference test result was determined by linear regression, and Spearman's rho correlation coefficient. Bland-Altman analysis was used to assess the difference between EDTA and ST results [17]. The mean difference [ST result - reference result] and the limits of agreement [mean ±1.96 standard deviations (S.D.)] were calculated and represented graphically in difference scatter plots. The non-parametric Wilcoxon signed-rank test and the paired *t*-test for continuous data were used to establish statistical significance of paired comparisons with an alpha value of 0.05 (two-sided test). Clinical agreement was assessed by calculating sensitivity, specificity, PPV and NPV with binomial exact 95% confidence intervals (CIs) using thresholds of 350 cells/μL and 500 cells/μL.

## Results

### Patient demographics

From 15 to 28 December 2011, paired venous blood samples were collected from 51 patients. The characteristics of the study population are summarized in Table 1. Participants had a median age of 34 years (IQR: 28 - 45) and 35 were female (68.6%). Twenty-one individuals were on WHO stage 1 (40.7%), 22 on stage 2 (43.3%) 5 on stage 3 (10.0%), and 3 on stage 4 (6.0%). CD4 testing was performed to determine ART eligibility in 13 individuals (25.0%) and for monitoring purposes in 38 individuals (75%), and the median CD4+ T cell count was 507.5 cells/μL (IQR: 271.75 - 744.5).

**Table 1:** **Participant characteristics**

Characteristic	Value
Total patients: N	51
Female: n, (%)	35 (68.6)
Age (years): median, (IQR)	34, (28 - 45)
WHO stage: n, (%)	
1	21 (40.7)
2	22 (43.3)
3	5 (10.0)
4	3 (6.0)
Reason for testing: n, (%)	
ART eligibility	13 (25.0)
ART monitoring	38 (75.0)
CD4+ T cells: median, (IQR)	507.5, (271.75 - 744.5)

### Comparison of CD4 on ST and EDTA

Twenty-eight sets of samples were sent to the laboratory at Birchenough Bridge Hospital (BBH), and the remaining 23 sets of samples were sent to the laboratory at Murambinda Mission Hospital (MMH). One pair of specimens in which the ST results differed markedly from the EDTA result was excluded from the analysis. In the excluded pair of specimens the Day 0 CD4+ T-cell result was 796 cells/μL in the reference specimen, and 468 cells/μL on the ST specimen. We assumed that this discrepancy was due to mismatching of specimens (i.e. comparing specimens from different patients) due to a labelling error. Due to a transient stock-out of CD4 reagents in one of the laboratories, only 19 ST samples were tested on Day 14.

**Figure 1:**
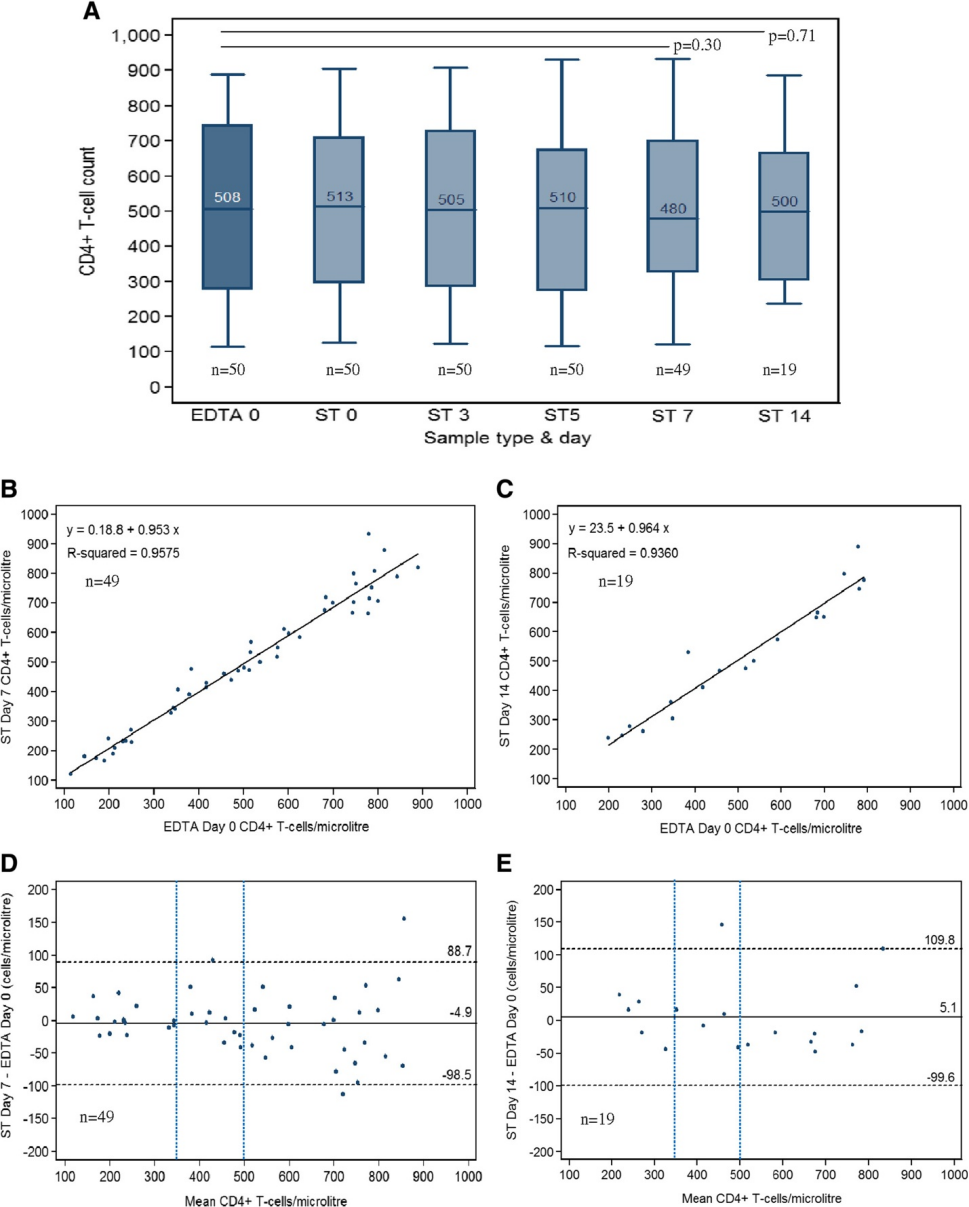
**Comparison of CD4 counts measured on ST (index test), at different time intervals, and EDTA at day 0 (reference test). A**: box-and-whisker plots displaying the median and upper and lower quartiles. **B** and **C**: scatter plots displaying correlation. **D** and **E**: Bland-Altman plots showing the mean difference (solid line) and 95% lower limits of agreement (dotted lines). Blue vertical dashed lines indicate CD4 T cells thresholds of 350 cell/μL and 500 cells/μL.

After exclusion of the one pair of samples, the median CD4+ T-cell count in the reference test (EDTA Day 0) was 508 cells/μL (IQR: 294 - 733). The median of CD4+ T-cell counts of ST samples were comparable to the median of the reference test and remained relatively constant over time and differences were not statistically significant on Day 7 (p = 0.30) and Day 14 (p = 0.71) (Figure 1A). The summary statistics for correlation and agreement between ST and EDTA is shown in Table 2. There was a strong correlation between absolute CD4+ T-cell counts in the ST samples and the reference test, with Spearman's rho 0.9778 on Day 7, and 0.9632 on Day 14 (Figure 1B and 1C). Bland-Altman analysis found a mean difference of −4.9 cells/μL with LOA -98.5 to +88.7 cells/μL on Day 7 (Figure 1D); and a mean difference of 5.1 cells/μL, and LOA of -99.6 to +109.8 cells/μL on Day 14 (Figure 1E).

**Table 2:** **Summary statistics for correlation and agreement between CD4+ T cells in ST samples measured on Day 7 and Day 14 (index test), and CD4+ T cells in EDTA samples measured on Day 0 (reference test)**

Test	N	Median	p-value (EDTA Day 0)	R^2^	Spearman's rho	Mean bias (95% CI)	Standard deviation of bias	Limits of agreement
CD4 ST Day 7	49	480	0.30	0.957	0.977	-4.89 (-18.34 to 8.54)	46.81	-98.52 to 88.72
CD4 ST Day 14	19	500	0.71	0.936	0.963	5.10 (-20.13 to 30.34)	52.35	-99.61 to 109.82
CD4% ST Day 7	46	24.15	0.17	0.936	0.948	-0.53 (-1.14 to 0.06)	2.02	-4.59 to 3.51

**Figure 2:**
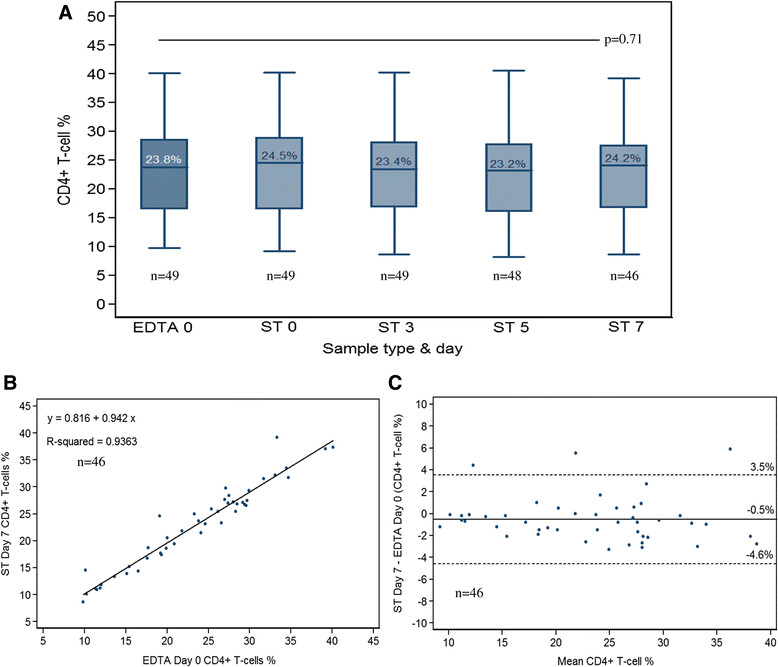
**Comparison of CD4% measured on ST (index test), at different time intervals, and EDTA at day 0 (reference test). A**: box-and-whisker plots showing the median and upper and lower quartiles. **B**: scatter plot displaying correlation. **C**: Bland-Altman plots showing the mean difference (solid line) and 95% lower limits of agreement (dotted lines).

The values of the CD4+ T-cell% also remained relatively constant over time and difference was not statistically significant on Day 7 (p = 0.17) (Figure 2A). There was a strong correlation between the CD4+ T-cell% in reference samples and the Day 7 ST samples (Spearman's rho 0.9778; Figure 2B). Bland-Altman analysis found a mean difference of -0.539%, and LOA of -4.6 to +3.5% (Figure 2C). Due to a transient stock-out of CD4 reagents in one of the laboratories, only 19 ST samples were tested on Day 14. CD4+ T-cell absolute counts were generated at Day 14 but the BD FACSCount flow cytometer did not produce CD4% results.

### Clinical agreement between CD4 measured on ST and EDTA

We calculated the agreement between the EDTA at day 0 and ST at day 7 and 14 using two clinically important cut-off values for CD4+ T cells that is currently used for initiating ART in Zimbabwe: 350 CD4 cells/μL and 500 CD4 cells/μL. Using a 350 cells/μL threshold, the sensitivity, specificity, positive predictive value (PPV) and negative predictive value (NPV) were all 100% on Day 7, and 83.3%, 100%, 100% and 92.9% on Day 14. Using a 500 cells/μL threshold, the sensitivity, specificity, PPV and NVP were 100%, 88.5%, 88.5% and 100% on Day 7 and 88.9%, 80.0%, 80.0% and 88.9% on Day 14. The sensitivity, specificity, PPV, NPV and misclassification rate are shown in Table 3. Using a threshold of 350 cells/μL, no ineligible patients would have been started on ART based on the Day 7 and the Day 14 ST results, and one eligible patient would have been denied ART based on the Day 14 ST results. Using a threshold of 500 cells/μL, three ineligible patients would have been started on ART based on the Day 7 results; two ineligible patients would have been started on ART based on the Day 14 ST results; and one eligible patient would have been denied ART based on the Day 14 ST results.

**Table 3:** **Clinical agreement and misclassification rates between CD4 measured on EDTA Day 0 (reference test) and CD4 measured on ST Day 7 and Day 14 (index test) at CD4 thresholds for ART eligibility of 350 cells/μL and 500 cells/μL**

EDTA Day 0	ST Day 7			Sensitivity: 100 (78.2 – 100); PPV: 100 (78.2 – 100)
	≤350	≤350	Total	Specificity: 100 (89.7 – 100); NPV: 100 (89.7 – 100)
≥350	15	0	15	Total misclassification rate: 0
≥350	0	34	34	Upward misclassification rate: 0
Total	15	34	49	Downward misclassification rate: 0
EDTA Day 0	ST Day 14			Sensitivity: 83.3 (35.9 – 99.6); PPV: 100 (47.8 – 100)
	≤350	≥350	Total	Specificity: 100 (75.3 – 100); NPV: 92.9 (66.1 – 99.8)
≤350	5	1	6	Total misclassification rate: 5.2
≥350	0	13	13	Upward misclassification rate: 5.2 (1/19)
Total	5	14	19	Downward misclassification rate: 0
EDTA Day 0	ST Day 7			Sensitivity: 100 (85.2 – 100); PPV: 88.5 (69.8 – 97.6)
	≤500	≥500	Total	Specificity: 88.5 (69.8 – 97.6); NPV: 100 (85.2 – 100)
≥500	23	0	23	Total misclassification rate: 6.1 (3/49)
≤500	3	23	26	Upward misclassification rate: 0
Total	26	23	49	Downward misclassification rate: 6.1 (3/49)
EDTA Day 0	ST Day 14			Sensitivity: 88.9 (51.8 – 99.7); PPV: 80.0 (44.4 – 97.5)
	≤500	≥500	Total	Specificity: 80.0 (44.4 – 97.5): NPV: 88.9 (51.8 – 99.7)
≥500	8	1	9	Total misclassification rate: 15.7 (3/19)
≥500	2	8	10	Upward misclassification rate: 5.2 (1/19)
Total	10	9	19	Downward misclassification rate: 10.5 (2/19)

## Discussion and conclusions

Results from this study show that specimens collected and stored in ST at room temperature are stable for up to 7 days without compromising the accuracy of absolute CD4+ T-cell counts or CD4+ T-cell% measurements. The Day 14 results were similar to the EDTA Day 0 results, suggesting that it may be possible to store ST specimens for longer than 7 days at room temperature (18°C to 25°C). However, the Day 14 results should be regarded as preliminary and interpreted with caution in view of the limited sample size (n = 19). The slightly better agreement of CD4 measured on ST on Day 7 can be seen by the negligible mean bias of -4.89 cells/μL and narrow 95% CI of mean bias (-18.34 to 8.54 cells/μL) versus 5.10 cells/μL and wider 95% CI of mean bias (-20.13 to 30.34 cells/μL) reflecting more variability in the agreement on Day 14. Using a threshold of 350 CD4 cells/μL we found 100% sensitivity and specificity, with no clinical misclassification of patients, of CD4 measured on ST on Day 7. The excellent sensitivity was maintained at a threshold of 500 CD4 cells/μL, but the specificity slightly dropped to 88.5%, and three patients (6.1%) would have been put on treatment based on the ST CD4 result. The slightly higher clinical misclassification seen at the 500 CD4 cells/μL threshold may be due to the intrinsic higher variability of the CD4 test at higher CD4 counts, as reported by others [18]. This can also be observed in the agreement scatter plot (Figure 1D) which revealed a higher variability, with a downward trend, when the CD4 count was higher, e.g. ≥500 cells/μL.

Our results corroborate the BD manufacturer's instructions which claim that specimens stored in ST at room temperature are stable up to 7 days without loss of accuracy. Our results are in agreement with those from Shott *et al*. [16] and Varro *et al.*[19] in which stability of specimens for CD4 testing was determined to be optimum at Day 6 and Day 5, respectively, at room temperature (change difference <10%); however, in these studies blood specimens were not tested after longer periods of storage. To our knowledge, the stability of blood specimens stored up to 14 days has not been described previously.

CD4 ST tubes offer several advantages in the decentralization of CD4 testing in remote settings. Firstly, specimen collection for CD4 testing can be done on the same day that patients attend the health facility for HIV testing or a follow-up clinic appointment, obviating the need to return to the clinic, thus reducing patient's transport costs and time spent on clinic visits. Rationalization of the patient's visit schedule is important, because multiple visits to the clinics increase the likelihood of pre-ART attrition [9],[10].

Secondly, ST tubes improve the stability and integrity of blood specimens, which is critical in the provision of high-quality CD4 results. Delays in specimen transportation with standard Vacutainer EDTA tubes may lead to spurious results due to the progressive decay of CD4 counts, especially at temperatures higher than 37°C [20].

Thirdly, ST tubes can simplify internal quality control procedures. Daily quality control entails running "normal blood samples" in conjunction with the commercial control beads, and laboratories usually rely on donors to obtain normal samples; this may prove difficult owing to the need to find a donor on a day-to-day basis. Normal blood samples collected in CD4 ST can be stored for several days and used multiple times for quality control purposes.

Despite the move towards initiating all patients who test HIV positive on ART, regardless of their CD4+ T-cell count, there is strong agreement among clinicians that a baseline CD4 test will remain necessary to guide clinical decisions about commencing prophylaxis and screening for OIs [21]. Given that CD4 testing remains important in ART programmes, it seems prudent that governments and stakeholders continue to invest in sample transport systems and strategies for the rapid delivery of laboratory results (mHealth), which will not only benefit HIV programmes but the health system as a whole. Recently, Kiyaga *et al.*[22] have shown the positive experience of Uganda in the development of an effective sample transport system to respond to the scale-up of early infant diagnosis (EID), the HUB system, whereby hubs feed into a centralized coordinated national system, thereby increasing efficiency and reducing costs and turnaround time of results, not only for EID but also for other laboratory services.

Although POC tests such as the Alere Pima CD4 have been shown to be an important intervention to improve retention in care prior to treatment initiation [11], costs may hamper its widespread implementation in the lowest-resource settings [12]. Experience from implementers indicate that the introduction of POC technologies require substantial human and financial resources for ongoing training, supervision, data management, quality assurance, instrument maintenance and supply control [23]-[25].

CD4 ST are more expensive than standard EDTA tubes ($0.7 per 4 ml tube and $0.4 per 2 ml tube, compared to $0.11 for standard 4 ml EDTA Vacutainer tubes). The higher cost of CD4 ST may serve as a deterrent to adoption of this technology.

Our study has several strengths; it was carried out under real-world, programmatic conditions, and samples were collected and transported from rural clinics to a central laboratory.

Some limitations in this study need to be acknowledged. Firstly, experiments were carried out in December during the rainy season so we were unable to assess the stability of blood samples at higher temperatures. Secondly, as the number of ST samples tested on Day 14 was relatively small (n =19), the results need to be interpreted with caution. And thirdly, most of the participants recruited for this study were having CD4 testing for monitoring purposes, rather than to assess their eligibility for ART, and therefore they are not representative of patients having CD4 testing to determine ART eligibility. This may limit the generalisability of our findings concerning PPV and NPV.

Our study confirms previous findings and provides further evidence that CD4 STs can be used as an alternative to standard EDTA tubes in settings where testing within 48 hours is not feasible. Results of this study may stimulate further research to compare the cost of POC CD4 technologies with improved sample transport systems coupled with use of STs and rapid reporting of results and the impact of different strategies on patient outcomes.

## Authors’ original submitted files for images

Below are the links to the authors’ original submitted files for images. Authors’ original file for figure 1 Authors’ original file for figure 2
