# Supplementary figures and images for: Opportunities to improve storage and transportation of blood specimens for CD4 testing in a rural district in Zimbabwe using BD vacutainer CD4 stabilization tubes: a stability and diagnostic accuracy study
Source: BMC Infect Dis. 2014 Oct 22;14:553. doi: 10.1186/s12879-014-0553-9 (PMC4209078; doi:10.1186/s12879-014-0553-9)

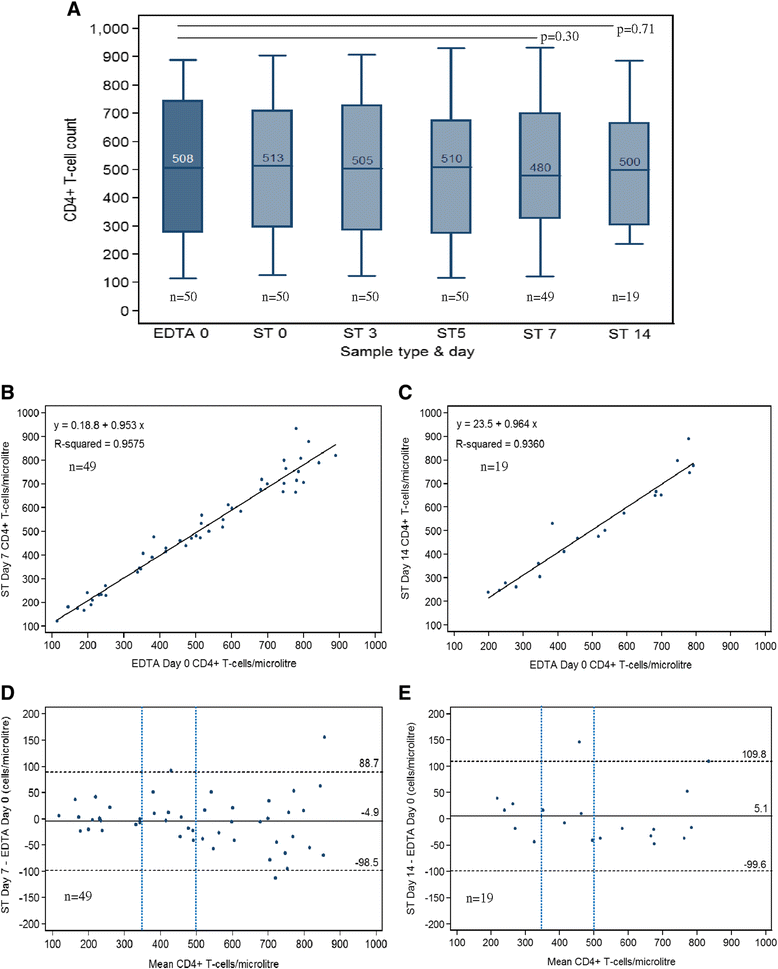

Supplement: Supplementary file 1 — Authors’ original file for figure 1 [file 12879_2014_553_MOESM1_ESM.gif]

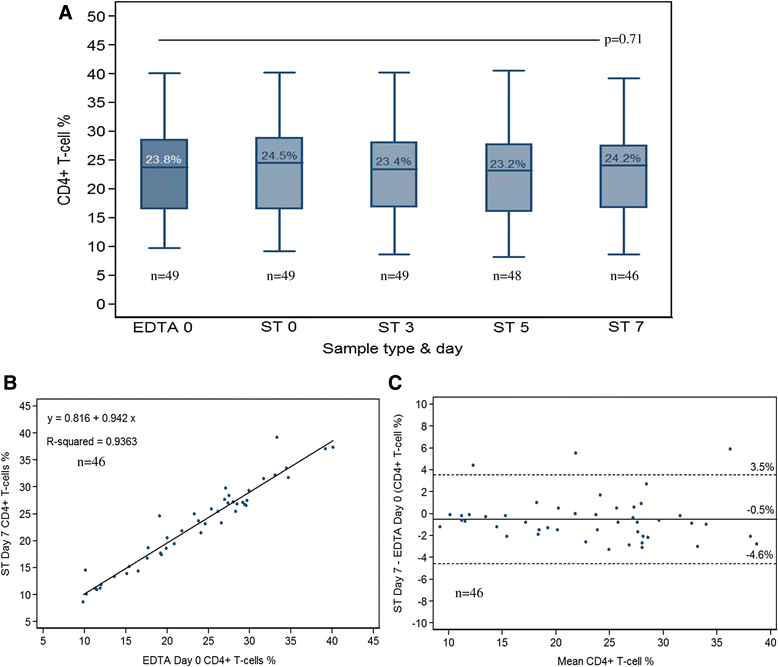

Supplement: Supplementary file 2 — Authors’ original file for figure 2 [file 12879_2014_553_MOESM2_ESM.gif]
